# Guest Editorial: Special Issue on Artificial Intelligence in E-Healthcare and M-Healthcare

**DOI:** 10.1155/2021/9857089

**Published:** 2021-12-02

**Authors:** Xingwang Li, Lingwei Xu, Thomas Aaron Gulliver, Han Wang

**Affiliations:** ^1^School of Physics and Electronic Information Engineering, Henan Polytechnic University, Jiaozuo 454000, China; ^2^Department of Information Science and Technology, Qingdao University of Science and Technology, Qingdao 266061, China; ^3^Department of Electrical and Computer Engineering, University of Victoria, Victoria BC V8W 2Y2, Canada; ^4^Faculty of Data Science, City University of Macau, Macau 999078, China; ^5^College of Physical Science & Engineering, Yichun University, Yichun 336000, China

## 1. Background

With the development of society and the changes in people's lifestyles, more chronic illnesses have become the world's biggest killers. According to the World Health Organization (WHO), ischemic heart disease, stroke, chronic obstructive lung disease, and lower respiratory infections have remained the top killers during the past decade in both developed and developing countries. Artificial intelligence (AI) has emerged as a promising technology in healthcare with many applications, including diagnosis, treatment, prevention, and medical payment systems. Recently, a great number of new information technologies have emerged to assist the applications of AI in healthcare, such as big data and mobile internet networks. Therefore, AI-based electronic (E)-healthcare and medical (M)-healthcare solutions are aimed at gaining information, processing it, and giving a well-defined output to the end-user (the physician, the patient, or the caregiver).

AI-based E-healthcare and M-healthcare have shown significant potential to achieve the goals and demands of medical treatment. Owing to the data-driven approach, AI has brought a paradigm shift from information fields to other applications, such as intelligent diagnosis and treatment, intelligent payment, intelligent drug research and development, and intelligent health management and diagnosis related groups. Consequently, many researchers in the information and communication technology field are focusing on AI for healthcare applications.

The prime aim of this Special Issue is to bring together researchers to report recent research in AI in healthcare and exchange new ideas with innovative technologies and solutions towards AI in E-healthcare and M-healthcare applications. The proposed submissions and presentations should be original and unpublished works.

The Special Issue is composed of 11 outstanding contributions. The topics of these articles are mainly concerned with AI technology for E-healthcare and M-healthcare. We believe that these articles will play a role in inspiring our readers.

## 2. Summaries of Accepted Articles

The first article “An Improved Double Channel Long Short-Term Memory Model for Medical Text” authored by Shengbin Liang et al. presented an improved Double Channel (DC) mechanism as a significant enhancement to Long Short-Term Memory (LSTM) to solve the triage and precise treatment of patients. The experimental results have shown that the DC-LSTM model proposed by the authors has significantly superior accuracy and ROC compared with the basic CNN-LSTM model.

The second article is “Stroke Lesion Detection and Analysis in MRI Images Based on Deep Learning” authored by Shujun Zhang et al. In the article, three categories of deep learning object detection networks including Faster R-CNN, YOLOV3, and SSD were applied to implement automatic lesion detection with the best precision of 89.77%. Meanwhile, statistical analysis of the locations, shapes of the lesions, and possible related diseases was conducted with valid conclusions. The research contributed to the intelligent assisted diagnosis and prevention and treatment of ischemic stroke.

The third article “Analysis on the Development Status of Coal Mine Dust Disaster Prevention Technology in China” authored by Hui Zhang et al. introduced the basic concepts, generation, distribution, and hazards of coal mine dust and analyzed the characteristics, applicable conditions, and use effects of various dust control measures such as ventilation dust removal and wet dust removal. The authors proposed specific prevention and control measures for related occupational diseases and discussed the development trend of dust prevention and control technology in the hope of providing guidance and reference for coal mine dust prevention and control.

The fourth article “Predicting Mental Health Problems with Automatic Identification of Metaphors” authored by Nan Shi et al. proposed a method for automatically detecting metaphors in texts to predict various mental health problems, specifically anxiety, depression, inferiority, sensitivity, social phobias, and obsession. They performed experiments on a composition dataset collected from second-language students and on the eRisk2017 dataset collected from Social Media. The experimental results showed that the proposed approach can help predict mental health problems in authors of written texts, and the proposed algorithm performs better than other state-of-the-art methods.

The fifth article “Cross-Database Micro-Expression Recognition Exploiting Intradomain Structure” authored by Yanliang Zhang et al. overcame this problem by exploiting the intradomain structure. Nonparametric transfer features were learned through intradomain alignment, while at the same time, a classifier was learned through intradomain programming. In order to evaluate the performance, a large number of cross-database experiments were conducted in CASMEII and SMIC databases. The comparison of results showed that this method can achieve a promising recognition accuracy and with high computational efficiency.

The sixth article “Efficient Algorithms for E-Healthcare to Solve Multiobject Fuse Detection Problem,” authored by Ijaz Ahmad et al., focused on the improved K-nearest neighbor (MK-NN) algorithm for electronic medical care to realize intelligent medical services and applications. The authors introduced modifications to improve the efficiency of MK-NN, and a comparative analysis was performed to determine the best fuse target detection algorithm based on robustness, accuracy, and computational time. Experimental results showed that the improved K-NN algorithm was the best model in terms of robustness, accuracy, and computational time.

The seventh article “ASC Performance Prediction for Medical IoT Communication Networks,” authored by Fagen Yin et al., investigated the secrecy performance of medical IoT communication networks. To improve the secrecy performance, the authors adopted a cooperative communication strategy. The authors also used the average secrecy capacity (ASC) as a metric, and the expressions are first derived. Then, a secrecy performance intelligent prediction algorithm was proposed. The extensive simulations were used to verify the proposed method. Compared with other methods, the proposed algorithm realized a better prediction precision.

The eighth article is “Clinical Effects of Form-Based Management of Forceps Delivery under Intelligent Medical Model,” authored by Siming Xin et al. In the article, patients with forceps delivery in Maternal and Child Health Hospital Affiliated to Nanchang University were divided into two groups: form-based patients from January 1, 2019, to December 31, 2020, were selected as the study group, while traditional protocol patients from January 1, 2017, to December 31, 2018, were chosen as the control group. The conclusions were that form-based management could help assess the security of forceps delivery comprehensively, as it could not only improve the success rate of the one-time forceps traction scheme but also reduce the incidence of maternal and neonatal adverse outcomes effectively.

The ninth article “Service Migration Policy Optimization considering User Mobility for E-Healthcare Applications,” authored by Xuhui Zhao et al., proposed a service migration solution based on migration zone and formulated service migration cost with a comprehensive model that captures the key challenges. The authors also formulated a service migration problem into Markov decision process to obtain optimal service migration policies that decide where to migrate in a limited area. The authors proposed three algorithms to resolve the optimization problem given by the formulated model. The results showed that the proposed service migration approach reduces the total cost by up to 3 times compared to no migration and outperforms the general solution in terms of the total expected reward.

The tenth article “Zero-Watermarking Algorithm for Medical Image Based on VGG19 Deep Convolution Neural Network,” authored by Baoru Han et al., proposed a robust zero-watermarking algorithm for medical images' security based on VGG19. The proposed algorithm utilized Hermite chaotic neural network to scramble the watermarking image for secondary protection, which enhanced the security of the algorithm. Compared with the existing related works, the proposed algorithm was simple to implement and could effectively resist local nonlinear geometric attacks, with good robustness, security, and invisibility.

The eleventh article is “A High-Sensitivity Method Based on Advanced Optical Waveguide Technology to Detect HBsAg,” authored by Pingping Xiao and Xiaoxiong Hu. A novel method for the detection of the hepatitis B surface antigen (HBsAg) at low concentrations, using the ultrahigh-order guided mode acting as the probe excited by a symmetrical metal-cladding waveguide, was proposed in the article. The measurement results indicated that this new method can precisely detect HBsAg at the concentrations in the lower region of the clinical gray area (i.e., below 20 ng/mL), the lower region of the current clinical gray area of HBsAg (below 20 ng/ml) can be measured, and the resolution can be reached (2 ng/mL).

